# Surface Functionalization of Nanofibers: The Multifaceted Approach for Advanced Biomedical Applications

**DOI:** 10.3390/nano12213899

**Published:** 2022-11-04

**Authors:** Deepak Kulkarni, Shubham Musale, Prabhakar Panzade, Ana Cláudia Paiva-Santos, Pratiksha Sonwane, Monika Madibone, Puja Choundhe, Prabhanjan Giram, Simona Cavalu

**Affiliations:** 1 Department of Pharmaceutics, Srinath College of Pharmacy, Bajajnagar, Aurangabad 431136, India; 2 Formulation and Development Department, Aculife Healthcare Pvt. Ltd., Sachana, Ahmedabad 382150, India; 3 Department of Pharmaceutical Technology, Faculty of Pharmacy of the University of Coimbra, University of Coimbra, 3004-531 Coimbra, Portugal; 4 REQUIMTE/LAQV, Group of Pharmaceutical Technology, Faculty of Pharmacy of the University of Coimbra, University of Coimbra, 3004-531 Coimbra, Portugal; 5 Department of Chemistry, Srinath College of Pharmacy, Bajajnagar, Aurangabad 431136, India; 6 Department of Pharmaceutical Sciences, University at Buffalo, The State University of New York, Buffalo, NY 14214, USA; 7 Dr. D. Y. Patil Institute of Pharmaceutical Sciences and Research, Pune 411018, India; 8 Faculty of Medicine and Pharmacy, University of Oradea, 410087 Oradea, Romania

**Keywords:** nanofibers, electrospinning, surface functionalization, biomedical applications

## Abstract

Nanocarriers are gaining significant importance in the modern era of drug delivery. Nanofiber technology is one of the prime paradigms in nanotechnology for various biomedical and theranostic applications. Nanofibers obtained after successful electrospinning subjected to surface functionalized for drug delivery, biomedical, tissue engineering, biosensing, cell imaging and wound dressing application. Surface functionalization entirely changes physicochemical and biological properties of nanofibers. In physicochemical properties, wettability, melting point, glass transition temperature, and initial decomposition temperature significantly change offer several advantageous for nanofibers. Similarly, biological properties include cell adhesion, biocompatibility, and proliferation, also changes by functionalization of nanofibers. Various natural and synthetic materials polymers, metals, carbon materials, functional groups, proteins, and peptides, are currently used for surface modification of nanofibers. Various research studies across the globe demonstrated the usefulness of surface functionalized nanofibers in tissue engineering, wound healing, skin cancers, melanoma, and disease diagnosis. The delivery of drug through surface functionalized nanofibers results in improved permeation and bioavailability of drug which is important for better targeting of disease and therapeutic efficacy. This review provides a comprehensive insight about various techniques of surface functionalization of nanofibers along with its biomedical applications, toxicity assessment and global patent scenario.

## 1. Introduction

Nanofibers have attracted considerable massive attention and emerged as novel nanomaterials due to potential physicochemical properties, surfactant free and, commercial potential [1,2]. Their advanced applications can be attributed to the ability to form network of highly porous mesh with notable interlinking amongst pores [3]. Nanofibers possess fiber diameter of less than 1000 nm and display excellent properties i.e., greater surface area to volume ratio, nanoporous surface morphology, greater drug loading, advanced physicochemical and biological properties [4]. The applications of nanofibers are not limited to drug delivery, biosensing, tissue engineering scaffolds and wound dressing [5].

Various methods for fabrication of nanofibers are summarized in Table 1, along with its advantages and disadvantages. Amongst different techniques used for the fabrication of nanofibers, the electrospinning method is the most extensively employed because it is considered as most acceptable, economic, and scalable technique for the production of nanofibers [6].

Electrospun nanofiber is a multifaceted approach and received greater attention owing to the diverse prospective applications for drug delivery carriers, biomedical devices, and tissue engineering scaffolds [7,8]. Electrospinning approach encompasses the application of an electric field to the polymer solution or polymer melt for the construction of polymer nanofibers. Nanofibers possessing precise surface properties are always desirable for various applications. Hence, surface functionalized nanofibers having specific surface properties are of interest which may extend their applications [9]. In addition, surface attributes may affect wettability, electrical conductivity, optical property, and biocompatibility. Several strategies, such as physical, chemical, and biological, have been applied for the design of surface functionalized nanofibers. Nanofibers’ surface modification involved plasma treatment, wet chemical method, surface graft polymerization, and co-electrospinning of surface-active agents and polymers [10]. Many bioactive molecules have been employed for the surface modification of degradable and non-degradable synthetic nanofibers for progressive biological as well as therapeutic applications [11]. Furthermore, nanofibers prepared by electrospinning technique using Poly (ε-caprolactone), poly (L-lactide-co-3-caprolactone) and poly (lactic co-glycolic acid) found promising for human body applications in tissues like bone, nerve, ligament, etc. and efficient scaffold for tissue engineering and drug delivery. Besides, other applications of electrospun nanofibers includes relevant to environmental protection, water purification, energy harvesting/conversion/storage, heterogeneous catalysis, encapsulation of bioactive species, surface coating, smart textiles, and regenerative medicine [12].

The various techniques employed for the characterization of surface functionalization nanofibers are scanning electron microscopy (SEM), transmission electron microscopy (TEM), Fourier transform infrared spectroscopy (FTIR), differential scanning calorimetry (DSC), Atomic force microscopy (AFM), X-ray diffraction (XRD), X-ray photoelectron microscopy (XPS), Energy-dispersive X-ray spectroscopy (EDAX), BET analysis and mercury porosimetry, etc. [13,14].

**Figure 1:**
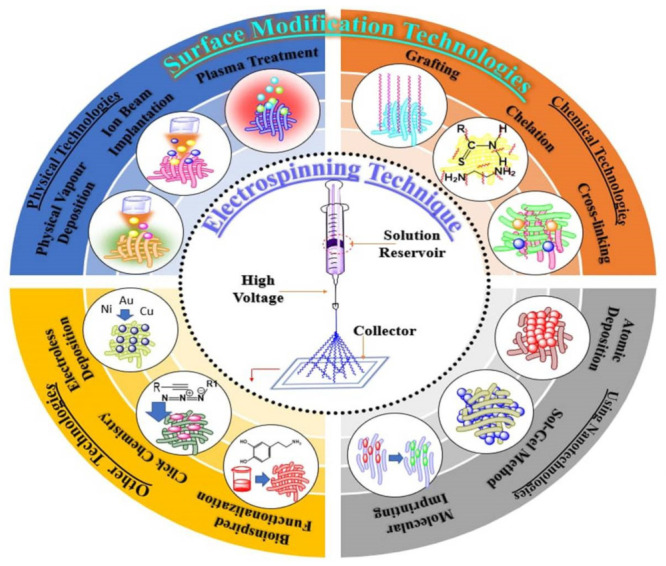
Surface modification techniques for nanofibers.

The extensively employed technique for the manufacture of nanofiber is electrospinning. It is considering as a simple, cost effectiveness and versatile method. These surface functionalized nanofibers have been particularly used in tissue engineering and wound healing because nanofibrous membranes resemble the natural extracellular matrix and that can foster the proliferation and migration of cells. Electrospun nanofibers can be used in wound healing due to its antibacterial activity, able to promote rapid hemostasis and having good biocompatibility to promote cell growth. There are various techniques employed for surface functionalization of nanofibers (Figure 1).

Considering several applications of the electrospun nanofibers, there is a need to focus on safety issues. Additionally, safety of the materials used for human application must be examined according to ICH guidelines. In vitro evaluation in animal models and clinical testing in humans should be undertaken to assess the toxicity profile of nanofibers after surface functionalization. Therefore, sufficient toxicity profile is required for human use in accordance with the standard regulatory guidelines [15].

The present review is focused to give detail information of electrospinning technique, methods of surface functionalization, reagents used for surface functionalization, and toxicity evaluation. Moreover, patent scenario and applications of surface functionalized nanofibers have also been discussed. This manuscript is the comprehensive literature providing valuable information on every aspect related to electrospinning and surface functionalization of nanofibers for advanced applications in the field of healthcare and biomedical sciences.

## 2. Electrospinning Technique

Electrospinning is a traditional technique used for the fabrication of the submicron to nanoscale size nanofiber with different morphological structure such as non-woven, core-sheath, porous, aligned nanofiber. This different morphology is used for different applications [16]. Fabrication process of nanofiber depends on the balance between surface tension of polymeric solution at the needle tip and applied external magnetic field. When critical threshold occurs, repulsive forces in solution overcomes the surface tension, charged jet travel from needle tip to collector that time solvent is evaporated from the polymeric solution, finally this jet deposited on the collector in the form of ultrafine nanofiber [17]. With reference to previous research studies, nanofibers are fabricated by using synthetic, semi-synthetic, and natural polymers. According to the study report, some polymers are difficult to produce electrospinning of polymeric nanofiber, especially natural polymer due to some limitation such as low molecular weight, weak mechanical properties as well as instability. These polymers require blending and surface functionalization with bioactive molecule, different chemicals moiety, and inorganic ions to make polymeric solution spinnable for development of nanofiber [18,19,20]. Alternation in simple electrospinning set up results in development of new techniques such as melt electrospinning, co-axial electrospinning, tri-axial electrospinning, needle less electrospinning, side-by-side electrospinning, centrifugal electrospinning or rotary jet electrospinning, multi-jet electrospinning, emulsion electrospinning, bubble electrospinning. Porous nanofiber prepared by using highly volatile solvent, on the other hand, for fabrication of aligned oriented nanofiber rotating drum collector set up is needed [21,22]. In this section, fabrication techniques and modified electrospinning set up for preparation polymeric nanofibers are summarized.

### 2.1. Melt Electrospinning

This is a melt-based technique used for the development of polymeric nanofiber from the melted form of polymer and also, use for the elimination of non-volatile organic solvent from polymeric nanofiber fabricating process. Melt electrospinning set up quietly analogous to traditional electrospinning set up, they consist of a spinneret, collector, and high voltage supply. In this electrospinning technique, the molten stream of the polymer is forcibly expelled through the spinneret and the polymeric nanofiber mechanically drawn to the grounded collector with continual flow where solidification is attained by the help of rapid cooling to form nanofiber scaffold for biomedical application. However, the individual diameter of nanofiber developed by this technique generally is larger than compared to nanofiber obtained from the traditional technique because of high viscosity of molten polymer [23,24]. Melt electrospinning technique is useful for the natural polymer like chitosan or starch, which need to dissolve natural polymer in organic solvent for fabrication of polymeric nanofiber [25,26]. In the literature reported authors successfully developed cardiac scaffold by melt electrospinning, of hydroxyl functionalized polyester, (poly (hydroxymethylglycolide-co-ε-caprolactone) [27].

### 2.2. Co-Axial Electrospinning

In recent years, co-axial electrospinning technique attracts to the researcher for formation of complex product of polymeric nanofiber for various biomedical applications. Advancement of this technique is beneficial for dual drug loading as well as sustained release application along with improving mechanical strength and functional properties without change in surface chemistry of polymeric nanofiber [28,29]. Additional advantage of this technique is controlling the internal structural morphology of core-shell nanofiber such as hollow, multi-channeled, fiber-in-tube, and tube-in-tube [30,31]. In this technique, polymeric nanofiber is fabricated by using two miscible and immiscible solvent systems. The drug is entrapped in the core region, and these core regions are completely cover by shell for protection of active pharmaceutical ingredient while extending its pharmaceutical delivery [32,33]. In another study, the authors fabricated core-shell nanofiber of poly (ether sulfone) and, poly(caprolactone) for bone tissue engineering by co-axial electrospinning, demonstrating enhance osteoblast and, chondrocytes differentiation [34]. Similarly, authors formulated gelatin and polycaprolactone nanofiber by co-axial technique in which polycaprolactone was encapsulated in the core, while gelatin incorporated in outer shell enhances the hydrophilicity of nanofiber mat [35].

### 2.3. Emulsion Electrospinning

To overcome some limitations of the co-axial or blend electrospinning technique, emulsion electrospinning emerged as a novel alternative approach for the fabrication of core-shell nanofiber by preparing core-sheath emulsified solution [36]. According to the former literature, this type of technique can be controlled by various parameters as compared to blend or co-axial electrospinning. Emulsified solution based on two or more types of phases contains functional material along with active pharmaceutical ingredient in the existence of an emulsifying agent. In the literature review report commonly, two types of emulsified solution are prepared for the electrospinning process includes, water-in-oil emulsion and oil-in-water emulsion. This emulsified solution was electrospun in specific manner the continuous phase is transformed into shell of nanofiber while droplet phase converted into core nanofiber in the presence of static electric field [37,38]. Emulsion electrospinning method is preferred over the polymer melting method as the non-melting polymers can also easily electrospun into nanofibers by this method. Furthermore, this technique has one limitation, polymeric solution with low interfacial tension does not perform properly [37]. This method can be applied for various functional material such as inorganic materials, natural, synthetic, semi-synthetic polymeric material for generation ultrafine nanofiber in various biomedical application [18,39]. In the literature report, the author formulates core-shell nanofiber based on polyvinyl alcohol blended with several polymers for cephalexin control release in wound healing applications. Study results demonstrate emulsion technique is an effective method for core-shell nanofiber formation as compared to co-axial approach [40]. Similarly, in study, polyvinyl and chitosan nanofiber developed by emulsification of cabreuva essential oil for the sustained release delivery in wound healing application [41].

### 2.4. Rotary Jet Spinning (RJS)

This technique is also termed as Forcespinning™, centrifugal spinning, and rotor spinning. RJS has some advantages to other electrospinning methods, such as eco-friendly, inexpensive, aligned nanofiber mats as compared to the conventional high-speed rotary drum collector method. The quality as well as size of nanofiber is easily tunable by the help of processing condition. The disadvantage for RJS is it requires high temperature for fabrication process [42,43]. In this technique, viscous polymer, whether from solution or melt put down into the spinning reservoir with tiny orifice. When angular velocity crosses the critical value, where the centrifugal force is larger than the capillary forces, that time polymer is ejected through orifice, same as conventional electrospinning technique [21]. This process does not use an electric field for elongation of polymer jet, instead of electric filed centrifugal force is applied for the jet elongation for fabrication of nanofiber. According to the study report, poor angular velocity leads formation of beads in nanofiber [44]. In the literature report, the author formulates nanofiber scaffold from polymeric solution, as well as from melt by using the RJS technique study results that demonstrates developed scaffolds has greater capability of high-level volume cell proliferation by using NIH3T3 fibroblast cells [45]. Similarly, in another report polycaprolactone developed with the mesenchymal stem cells study results demonstrates stem cells shows a cell proliferation to depth 250 µm, enough for fabrication of 3D nanofiber scaffold [46].

## 3. Methods of Surface Functionalization of Nanofibers

Several methods reported in the literature for surface functionalization of the nanofibers such as physical method include plasma treatment, physical vapor deposition, and ion beam implantation. Surface functionalization using chemical technologies involves surface grafting, cross-linking and chelation. These methods suffer from potential drawback and are not optimal. In the physical method, adsorption binding forces are weak, whereas chemical immobilization involves a harsh reaction condition, such as use of organic solvents, high temperature, and prolonged reaction time, which generate toxic allergens, cause degradation of material this method not suitable for biomedical application. Table 2 illustrates the various surface functionalization techniques with advantages and disadvantages.

### 3.1. Surface Functionalization Using Physical Technologies

#### 3.1.1. Plasma Treatment

**Figure 2:**
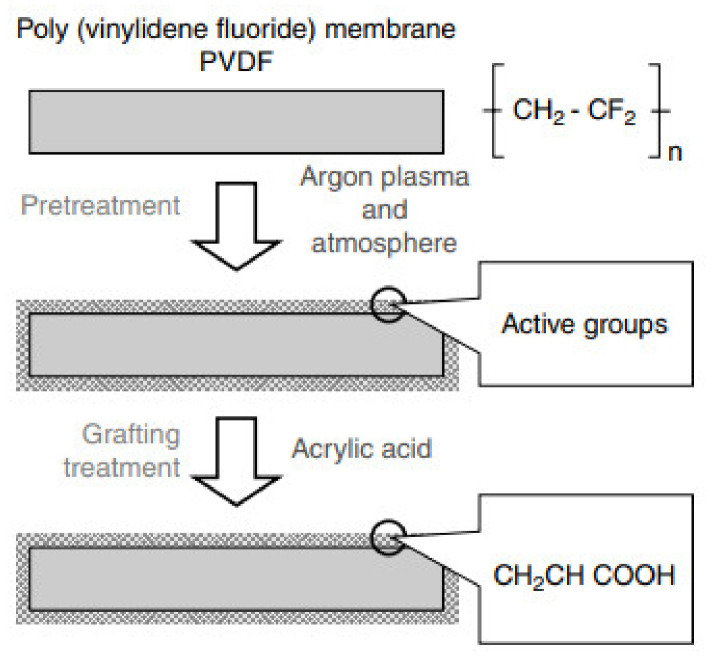
Pre-treatment and grafting on PVDF nanofiber membrane [49]. Adapted with permission from Ref. [49]. 2012, Elsevier.

The surface chemical composition of nanofibers is easily changed by plasma treatment method. After the plasma treatment, the change in the surface wettability and biological properties is reported in the literature. Plasma source selection is important for plasma treatment is based on its diverse functional group incorporated into nanofibers. Plasma source should be of sufficient strength to reach target surface, and covalent immobilization of bioactive molecules achieved. The surface hydrophilicity of nanofibers increases by air or argon plasma treatment [18]. Plasma treatment with discharge gas argon, ammonia, and helium on the surface of PCL preserved surface morphology of nanofibers and surface wettability significantly changes as compared with plasma un-treated nanofiber [47]. Plasma treatment is eco-friendly, clean, and environmental approaches to modify surface of nanofibers without affecting bulk properties. The surface of polyamide electrospun nanofibers modified by the cold gas plasma treatment results indicated an entire change in surface physical and chemical features. Plasma treatment for surface modification provides great potential for medical devices, biomaterial, and biosensor applications [48]. As shown in Figure 2, nanofibers of PVDF Poly (Vinylidene fluoride) on treatment with argon plasma and atmosphere converted to active functional group which further surface grafted with acrylic acid [49].

#### 3.1.2. Physical Vapor Deposition

New technique in coating of nanofibers surface by physical vapor deposition uses nitrogen, oxygen, and acetylene, etc. These gases introduced into chamber during deposition for surface coating. It involves evaporation and metallic sputter coating of surface of nanofibers [50].

#### 3.1.3. Ion Beam Implantation

The surface of polymeric nanofiber modified with ion beam implantation method. The modification with nitrogen ion treatment introduced amine or amide functionality on nanofibers surface. These functionalities on surface of nanofibers promote cell compatibility. This method has advantageous of controllability and reproducibility of results for surface coating. In the literature, PVA Poly (vinyl alcohol) nanofibers treated with ion beam implantation to study the mechanical properties [51].

### 3.2. Surface Grafting, Cross-Linking and Chelation

The surface modification by chemical method includes surface grafting, cross-linking, and chelation, etc.

#### 3.2.1. Grafting

**Figure 3:**
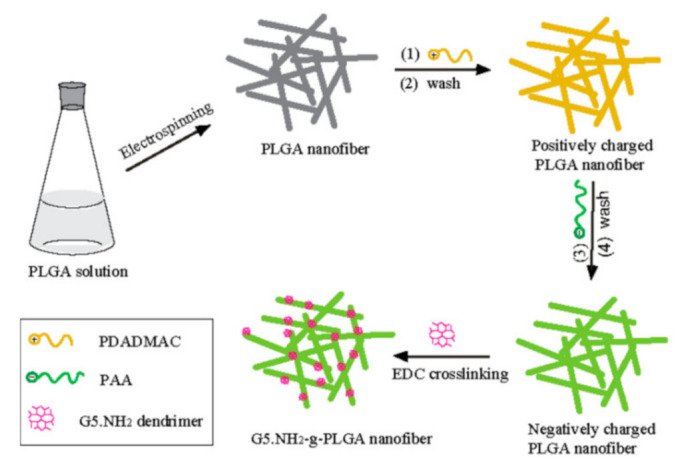
Preparation of G5·NH_2_-g-PLGA Nanofibers [52]. Adapted with permission from Ref. [52]. 2020, American Chemical Society.

PLGA electrospun nanofibers of uniform morphology pre-treated with positive charge diallyl dimethyl ammonium chloride and negatively charge Poly (acrylic acid) then immobilized of poly(amidoamine) (PAMAM) dendrimers(G5·NH_2_) by EDC cross coupling reaction on PLGA surface. First, carboxy group of Poly (acrylic acid) cross linked with amino group of PAMAM (G5·NH_2_) dendrimers, which further grafted to PLGA nanofibrous scaffold for gene delivery, as shown in Figure 3 [52].

#### 3.2.2. Cross Linking

**Figure 4:**
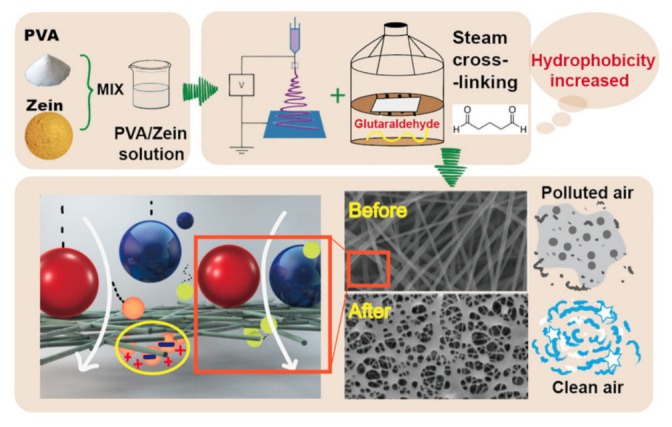
Schematic preparation and design of the multi-functional nano-filter based on hydrophobic cross-linked zein nanofibers Adapted from with permission Ref. [54]. 2020, Elsevier.

In the cross-linking strategy, various grades of chitosan nanofibers with different molecular weight polymeric solution converted into schiff base functionality of nanofibers by novel method with crosslinked glutaraldehyde vapor treatment. This cross-linked chitosan shown increase solubility as compared to no cross-linked nanofibers. The residue of the cross linker remained in nanofibers affect biocompatibility and toxicity of nanofibers. The series of available across linking agent is re epichlorohydrin, resimene, glutaraldehyde, and gepinin, etc., [53]. In the literature, zein based nanofibers electrospun with Poly (vinyl alcohol) and cross linking are performed with glutaraldehyde, as shown in Figure 4. The crosslinking of zein nanofibers cause significant improved in the adsorption capacity of nanofibers [54].

#### 3.2.3. Chelation

**Figure 5:**
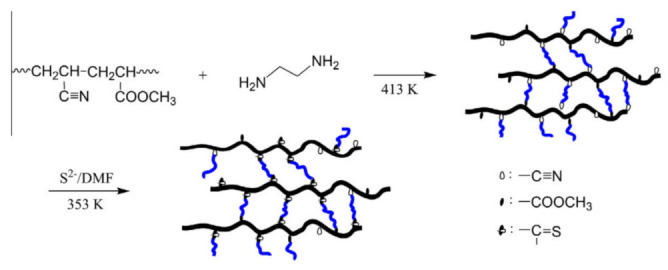
The synthetic scheme of thioamide-group chelating nanofibers based on PAN nanofibers [55]. Adapted with permission from Ref. [55]. 2013, Elsevier.

The Polyacrylonitrile (PAN) electrospun nanofibers obtained by electrospinning were subjected to chemical modification by cross-linking with diamine, ethylene glycol, and thioamide. These nanofibers can be efficiently explored for biomedical application. The schematic presentation for chelation nanofiber as shown in Figure 5 [55].

### 3.3. Electroless Deposition

Polyamide 6 polymeric solution electrospun functionalized by electroless deposition of (oxygen) O_2_ low plasma treatment was applied to replace routein roughening conventional process with sulphuric acid and potassium dichromate. In this work electroless deposition of copper on uniformly cover individual nanofibers surface for significantly change in surface morphology and conductivity observed and high surface area of Polyamide 6 nanofibers retained intactly. Similarly, nickel, gold, and copper used in the literature to give uniforms thickness of 160–400 nm [56].

### 3.4. Bioinspired Surface Functionalization

**Figure 6:**
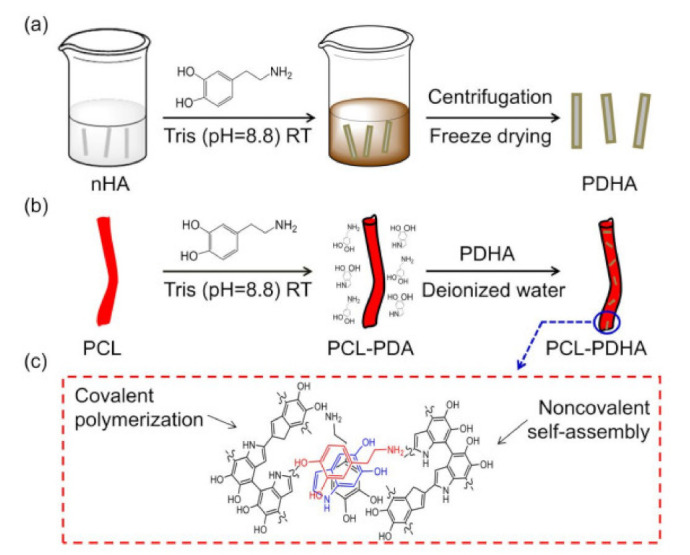
A schematic diagram showing bioinspired surface functionalization of electrospun PCL nanofibers for improving osteogenesis: (**a**) Preparation of PDHA; (**b**) Preparation of PCL-PDHA; (**c**) PDA formation mechanism based on both covalent polymerization and noncovalent self-assembly [57]. Adapted with permission from Ref. [57]. 2018, American Chemical Society.

The bioinspired surface functionalization of nanofibers was performed by surface coating approach. Surface coating is versatile and universally accepted method based on dopamine. Nanofibers electrospun with polymers containing non-surface-active group subjected to surface coating, as other surface functionalization methos not suitable. In the literature, PCL Poly(€-caprolactone) based electrospun nanofibers surface functionalized with n-Hydroxyapatite with aid of natural bioadhesive dopamine. In former research studies, the dopamine is reported as bioadhesive agent due to oxidation its catechol functional group under neutral or alkaline conditions results dopaminequinone which subsequently deposited in the form of stable durable film, as shown in Figure 6. The film thickness and stability easily tuned with concentration of dopamine. The dopaminequinone further converted to polydopamine by covalent polymerization and non-covalent self-assembly [57]. Similarly, dopamine used for bioinspired immobilization of bovine serum albumin for bilirubin adsorption and growth factor immobilization for repairing scars [58,59].

### 3.5. Click Chemistry

**Figure 7:**
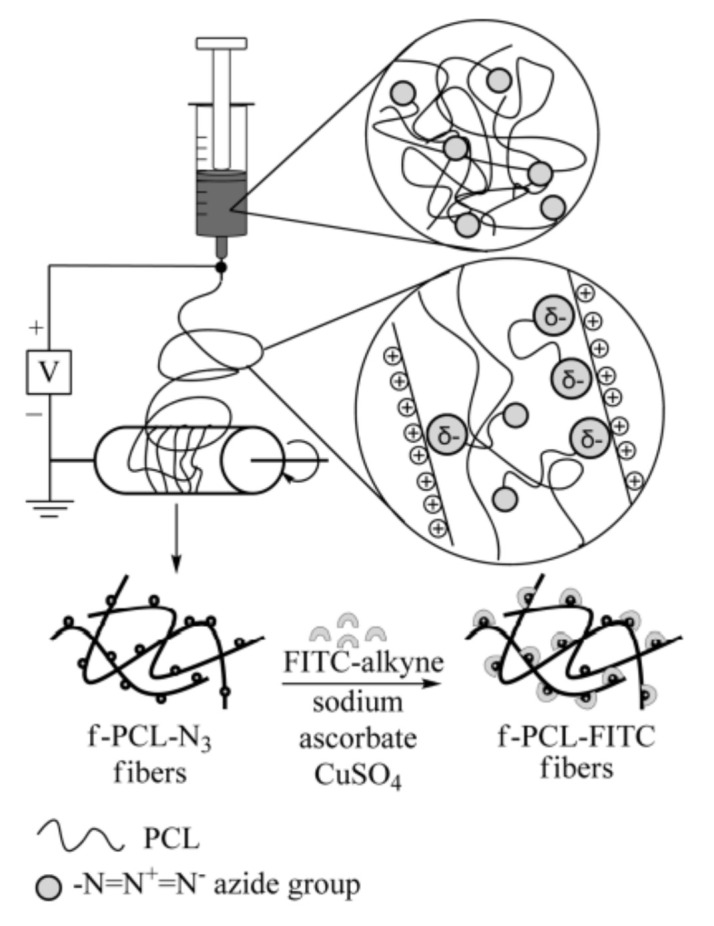
Schematic representation of the electrospinning setup with illustrated electrostatic attractions between negatively polarized azides (δ-) and positively charged surface during the electrospinning, as well as click reaction of f-PCL-N_3_ [61]. 2012, American Chemical Society. Adapted with permission from Ref. [61].

Click reaction involves reaction between azide and alkyne catalyzed by copper so called copper(I)-catalyzed azide-alkyne cycloaddition (CuAAC). The click reaction catalog contains important chemical reactions thiol-ene radical reaction, oxime ligation, Michael addition, and CuAAC, etc. It has been reported in the literature for bioconjugation, drug -polymer conjugation, and surface functionalization. These reactions are selective, efficient, and robust. In this reaction aqueous solvent used, ambient temperature, and high yield obtained. The combination of electrospun nanofiber of poly (ester urea) with pendant alkyne, azide, alkene clickable group and model reaction with fluorescent probe indicates presence of the clickable group on the surface of the nanofibers [60]. The desired biomolecules (peptides, protein, carbohydrate, aptamer, antibodies, and growth factor, etc.) decorated or immobilized on surface by click reaction. The functionalized nanofiber of Poly(€-Caprolactone) with azide group on surface of nanofibers obtained by electrospinning on click reaction with FITC-alkyne results in the surface functionalization of nanofibers for in vitro cell imaging via fluroscence shown in Figure 7 [61].

### 3.6. Surface Functionalization Using Nanotechnologies

#### 3.6.1. Sol-Gel Method

In sol-gel method modification of surface of electrospun nanofibers results in change in physical and chemical properties. It is reported in the previous literature that Poly (acrylonitrile) nanofibers functionalized by sol-gel method for deposion of zinc oxide. Poly (acrylonitrile) used as a substrate for functionalization of zinc oxide. Sol-gel method is simple and economic method used for surface deposion, etc., [62,63].

#### 3.6.2. Atomic Deposition

**Figure 8:**
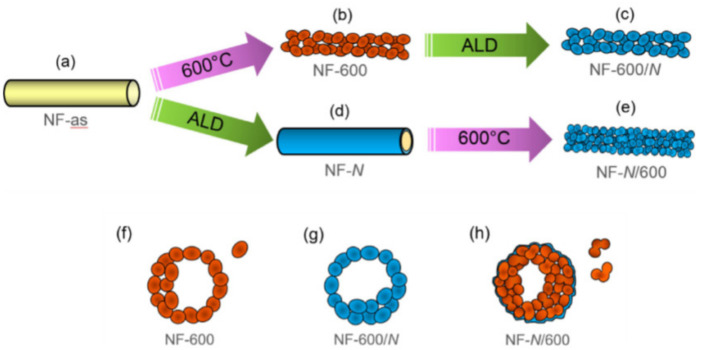
Schematic description of the experimental procedure followed to synthesize the two NF sets and of their morphology. NF600/N (**c**) are produced through the sequence HT-ALD, namely, operating the SiO_2_-coating via ALD over the α-Fe_2_O_3_ NFs (**b**) obtained by calcination in air at 600 °C of the as-spun PAN/FeAc_2_ NFs (**a**). Vice versa, NF-N/600 (**e**) are produced via calcination of the SiO_2_-coated PAN/FeAc_2_ NFs (**d**), that is, through the sequence ALD-HT. (**f**–**h**) Sketch of the NF cross section and of the α-Fe_2_O_3_ grain shape (the different grain shape and packing are responsible for the difference in the texture of NF-600(/N) and NF-N/600) [64]. Adapted with permission from Ref. [64]. 2020, American Chemical Society.

Atomic deposition on nanofiber surface is efficient method to obtained nanofibers with well-defined surface for specific applications. Poly(acrylonitrile) polymeric solution electrospun nanofibers dope with silver nanoparticles by in situ reduction in silver using N_2_H_5_OH aqueous solution. In the literature SiO_2_-Coated α-Fe_2_O_3_ nanofibers electrospun, calcinated and subjected to atomic layer deposion as shown in Figure 8 [64].

#### 3.6.3. Layer-By-Layer (Lbl) Deposition

Polymeric solution of chitosan deposited on carbon nanofibers previously electrospun. The deposition depends on the interaction of positively charged chitosan and negatively charged carbon nanofibers. Robotics dip coating method is used for layer-by-layer deposition [65].

#### 3.6.4. Molecular Imprinting

**Figure 9:**
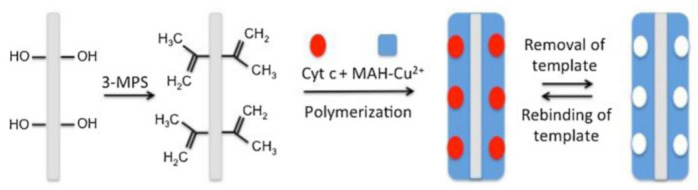
The schematic representation of the preparation method of cytochrome c imprinted bacterial cellulose nanofibers (Cyt c-MIP NFs). Before polymerization procedure, bacterial cellulose nanofibers were reacted with 3-MPS. The prepolymerization complex of metal chelation monomer, MAH, and template protein, Cyt c was prepared and polymerized onto the activated bacterial cellulose nanofiber surface. Then Cyt c molecules were removed leaving highly specific recognition sites complementary to Cyt c molecules [66]. Adapted from with permission Ref. [66]. 2015, Elsevier.

According to a study, bacterial cellulose nanofibers reacted with 3-methacryloxypropyltrimethoxysilane at 80 °C and washed with methanol and water. In next step metal chelation monomer complex N-Methacryloyl-(L)-histidinemethylester-Cu(II)-Cyt c solution prepared treated with bacterial cellulose nanofibers as shown in Figure 9. There molecular imprinted nanofibers used for biosensor, filtration, and biosepration [66].

### 3.7. Surface Functionalization Using Biotechnology

**Figure 10:**
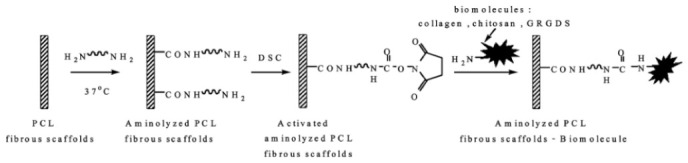
Chemical pathway for the immobilization of different biomolecules, such as collagen (i.e., marine collagen, COLM; type I collagen, COLI; type IV collagen, COLIV), chitosan (i.e., CS15, Mw) 15,000 g mol^−1^; CS83, Mw) 83,000 g mol^−1^), or GRGDS peptide (i.e., H-Gly-Arg-Cly-Asp-Ser-OH), on the surface of the electrospun PCL fibrous scaffold [67]. Adapted with permission from Ref. [67]. 2009, American Chemical Society.

Immobiliztion of bioactive molecules on the surface of electrospun nanofibers in order to obtain biologically functionalized nanofibers for drug delivery, tissue engineering, and biosensor application is one of the prime types of research reported in the literature. PCL Poly(€-caprolactone) nanofibers treated with hexamethylenediamine to introduce amine functionality on their surface then various biomolecules such as chitosan, and collagen immobilized using various coupling agents as shown in Figure 10 [67].

## 4. Reagents Used for Surface Functionalization

### 4.1. Fe_2_O_3_ (Ferrous Oxide/Iron Oxide)

Iron oxides and oxyhydroxides are vast in nature and play critical role in lots of biological tactics. Cai et al., fabricated pure polyamide 6 (PA6) nanofiber & PA6/organically altered montmorillonite (O-MMT) compound nanofibers by electrospinning, and further coated with nanosized iron oxide by magnetron sputter process. The energy dispersive X-ray analysis (EDX), X-ray photoelectron microscopy (XPS) and Atomic Force Microscopy (AFM) confirmed the modified surface morphology of compound nanofiber before & after sputter coating process. The resulting surface modified nanofibers showed improved thermal stability [68].

### 4.2. Gelatin

Gelatin is normally used as a gelling agent in meals, beverages, medicines, drug and nutrition pills, photographic films, and papers, and superficial. It’s having the notable utilization in surface modification of nanomaterials. Zuwei et al., altered the surface of electrospun poly (caprolactone) (PCL) nanofibers to alleviate the compatibility and spreading of endothelial cells (ECs). This gelatin grafted surface modified PCL nanofibers (PCL NF) showed efficient application in tissue engineering [69].

### 4.3. Silver

Silver is one of the prime metals used for fabrication of various nanomaterials. Silver is also used for surface functionalization of nanofibers. Nejad et al., prepared Mussel-inspired nanofibers decorated with silver nanoparticles for wound dressing. Nanofibers coated with silver nanoparticles (AgNPs) demonstrate significant antibacterial potential. The in vitro and in vivo studies also endorsed the usefulness of silver in coating or functionalization of nanofibers [70,71].

### 4.4. Plasma Treatment (Ar or O_2_ Gas)

Plasma treatment is one of the techniques used for surface modification of nanofibers and very well reported in the former literatures also. Neves et al., reported use of plasma treatment at low-power density for fabrication of electrospun polycaprolactone nanofibers (NFMs) for tissue engineering applications and making them more appealing to cells. X-ray Photoelectron Spectroscopy (XPS) determined the increase in oxygen-containing groups (-C=O and -OH) on the surface of plasma-treated nanofibers. Three cell types are used to study the effect of the modifications. All the three cell lines showed increased cellular connectivity after surface modification [72].

### 4.5. Graphene Oxide-Silver

**Figure 11:**
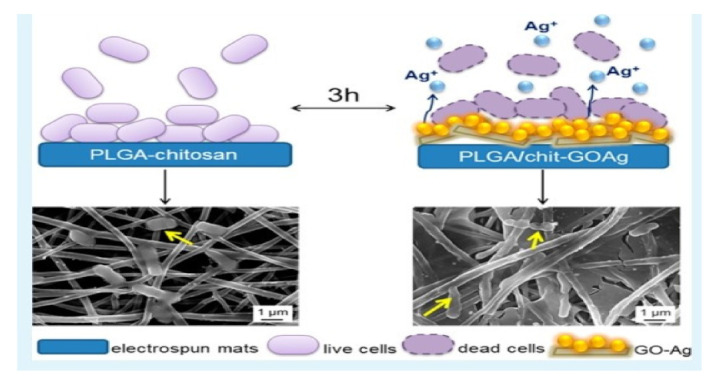
Preparation process of electrospun biopolymer nanofiber mats functionalized with graphene oxide-silver nanocomposites [73] Adapted with permission from Ref. [73]. 2015, American Chemical Society.

**Figure 12:**
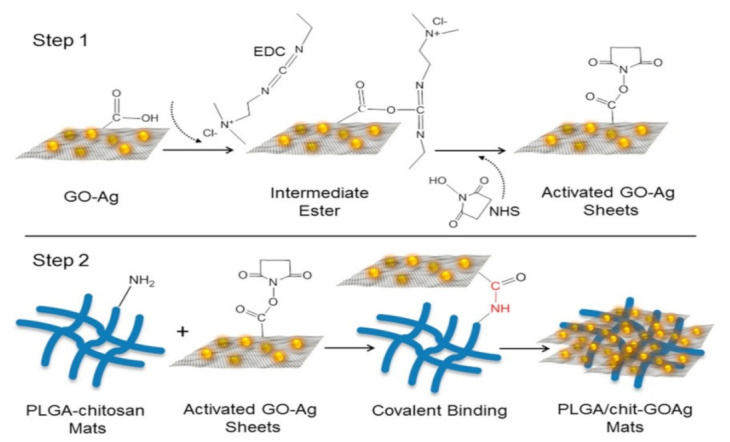
Schematic diagram showing the protocol for covalent binding of GO-Ag nanocomposite to the PLGA-chitosan nanofibers surface. Step1: the native carboxylic groups on GO sheets react with EDC and NHS to form an intermediate activated ester at pH 5.0 (MES buffer). Step 2: the native free amine groups present in the PLGA-chitosan nanofibers react with the intermediate ester, leading to the formation of a stable amide bond which covalently links the GO-based materials with the surface of the electrospun mats [73]. Adapted from with permission Ref. [73]. 2015, American Chemical Society.

Graphene oxide is a two-dimensional carbon material. It is composed of carbon atoms organized in a hexagonal lattice, which are linked together to form a regular two-dimensional sheet. Faria et al., reported the preparation of Graphene oxide-silver functionalized antimicrobial fiber mats produced by electrospinning with an antimicrobial property. In this study, researchers successfully developed electrospun mats that are functionalized with graphene oxide-silver nanocomposites. The binding of the graphene-based nanocomposite sheet to the fiber surface was examined using scanning electron microscope and transmission electron images. The PLGA-chitosan mats with GO-Ag-nanocomposites were able to effectively inhibit the gram-negative and gram-positive bacteria (Figure 11 and Figure 12) [73].

### 4.6. ZnO-Ag

Zinc oxide- and silver-based nanomaterials are also used for surface modification of nanofibers which resulted and significant efficiency. Hota et al., reported Surface functionalization of electrospun PAN nanofibers with ZnO-Ag heterostructure nanoparticles. In this research, surface functionalization of electrospun PAN nanofibers with ZnO-Ag heterostructure nanoparticles was catalyzed by various chemical pathways. The functionalized nanofiber showed antibacterial property. In the inhibition study it was shown that these surface modified nanofibers are excellent antimicrobial agents against E.coli and micrococcus leuteus [74,75].

### 4.7. Nano-Hydroxyapatite (nHA)

**Figure 13:**
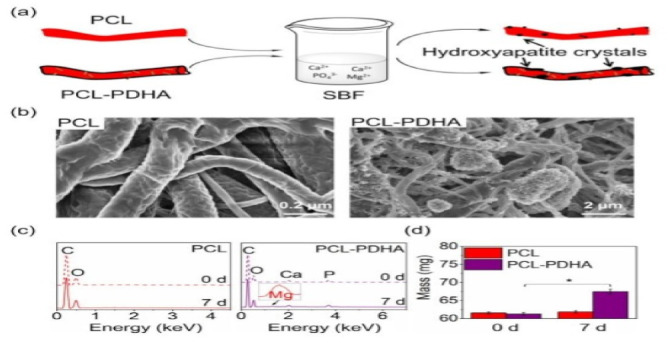
In vitro biomineralization of PCL and PCL-PDHA nanofiber membranes in SBF: (**a**) Schematic diagram of the biomineralization process of PCL and PCL-PDHA; (**b**) SEM images of biomineralized PCL and PCL-PDHA on day 7; (**c**) EDS analysis of PCL and PCL-PDHA before and after biomineralization; (**d**) Comparison of the amount of biomineralization product of PCL and PCL-PDHA, * *p* < 0.05 [57]. Adapted with permission from Ref. [57]. 2018, American Chemical Society.

Nanohydroxyapatite is a calcium-containing chemical compound. It is used to connect nanofibers to scientific labs for research into new potential treatments. Zhang et al., reported a bioinspired surface-functionalization approach to improve the osteogenesis of electrospun polycaprolactone (PCL) nanofibers. In this study, a versatile and effective approach was suggested to improve PCL nanofibers using bioactive nanohydroxyapetite using dopamine as an efficacious bioadhesive agent. In addition to woven, weft/interweave and PCL/PDHA copolymer nanofibers, PCL-PDHA blends significantly improved the bioactivity of nanofibers (Figure 13). The in vitro cell tests indicated the improved osteogenesis and biomineralization capabilities after functionalization [57,76].

### 4.8. Collagen Coating

Collagen is the most abundant structural protein in animals. Collagen is also used for surface modifications of electrospun nanofibers [77]. Zhang et al., reported characterization of the surface biocompatibility of the electrospun PCL—collagen nanofibers using fibroblasts. Nanofibers were made with a rough coating (i.e., PCL—collagen core-shell nanofibers in the form of a core-shell structure). This nanofibrous matrix was also prepared using the coaxial electrospinning technique by soaking PCL nanofibers with collagen. Coating with collagen on PCL nanofibrous matrix definitely favored cells proliferation, and therefore the efficiency is coating means dependent. In contrast, the roughly collagen-coated PCL increased only by 5.5% (2 days), 11.0% (4 days) and 21.0% (6 days). The collagen-r-PCL nanofibers encouraged cell migrations inside the scaffolds were indicated by SEM observation. Consistent with the given data and findings, the Collagen-r-PCL nanofibers might be used as novel functional biomimetic nanofibers toward achieving magnificent integration between cells and scaffolds for tissue engineering applications [78].

### 4.9. Polyelectrolyte: Poly (Acrylic Acid) (PAA), Chitosan (CS) and Polydiallyl Dimethyl Ammonium Chloride (pDADMAC)

Polyelectrolytes properties are virtually like electrolytes and polymers and are generally known as polysalts. Polyacrylic acid is electrolyte that is soluble in binary compound media at neutral pH. Schiffman et al. reported Polyelectrolyte-functionalized nanofiber mats to collection and inactivation of Escherichia coli. In this study, the cellulose nanofiber mat was functionalized with three polyelectrolytes: poly (acrylic acid) (PAA), Chitosan (CS), and polydiallyldimethylammonium chloride (pDADMAC). The study demonstrated that, the collection cellulose nanofiber mats can be tailor-made via a facile polyelectrolyte functionalization process. While the minimum conc. Of polycations needed to inhibit *E. coli* K12 was 800 microgram/mL for each CS and pDADMAC, as soon as immobilized, pDADMAC—functionalized nanofiber mats exhibited a higher inactivation of *E. coli* k12 (~97%) [79].

### 4.10. Avidin

**Figure 14:**
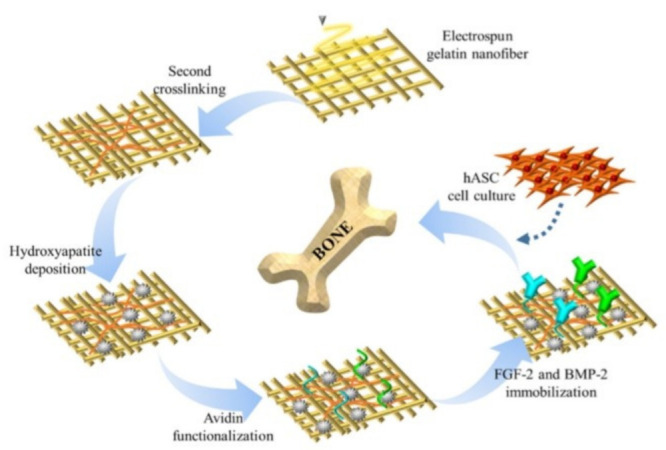
Schematic of fabrication process for FGF-2/BMP-2-immobilized hydroxyapatite/gelatin nanofibrous scaffold for bone tissue regeneration [80].Adapted with permission from Ref. [80]. 2020, Elsevier.

Avidin is tetrameric biotin-binding macromolecule created within the oviducts of birds, reptiles and amphibians and deposited in the whites of their eggs. Hydroxyapatite is a vital ingredient of traditional bone and teeth. Hydroxyapatite provides rigidity to bones and teeth. Hansoo Park et. Al. reported surface functionalization of dual growth factor on hydroxyapatite-coated nanofibers for bone tissue engineering. A scaffold of porous gelatin nanofibers were fabricated by electrospinning and coated with hydroxyapatite employing a simulated liquid body substance solution, and surface functionalized with avidin to facilitate binding with biotinylated growth factors, namely bone morphogenetic protein-2 (BMP-2) and fibroblast growth factor-2 (FGF-2) at different ratios, to improve bone growth and to imitate the function of natural extracellular matrix for sustained release of multiple growth factors (Figure 14). The hydroxyapatite nanofiber coating, as confirmed by the increased expression of osteogenic gene markers. Comparisons of the factor release profiles with those of the physical adsorption showed that avidin-biotin conjugation was potent for sustained release with controlled multiple proliferation delivery for bone tissue engineering [80].

### 4.11. Glutaraldehyde

**Figure 15:**
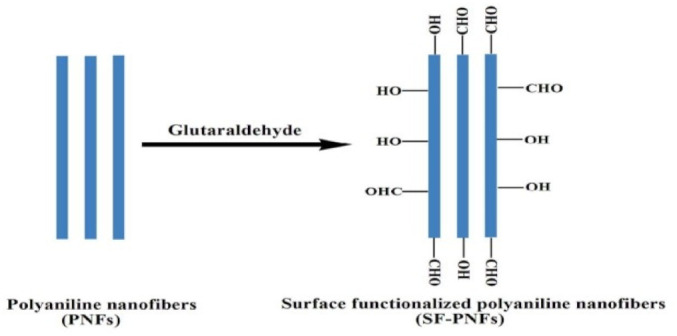
Schematic representation of surface functionalization of PNFs by glutaraldehyde [81]. Adapted with permission from Ref. [81]. 2015, Royal Society of Chemistry.

Glutaraldehyde, it is a disinfectant, medication, preservative, and also a fixative. Kumar et. Al. reported synthesis of Polyaniline (Pani) nanofibers by dilute polymerization method and functionalized by 1% glutaraldehyde solution in Phosphate buffer solution (PBS) (pH = 7.4). Functionalization enhanced the thermal stability of polyaniline nanofibers (PNFs), and which was further confirmed by thermo gravimetric analysis. After surface functionalization of PNFs the significant increase in percentage of cell viability was found with MTT assay of human peripheral blood mononuclear cells (PBMC). Very less haemolysis activity of surface functionalized polyaniline nanofibers (SF-PNFs) was revealed by Membrane stability test. It also indirectly indicates the improvement in antioxidant property of PNFs after surface functionalization. Non-cytotoxic effect of SF-PNFs can make it a biocompatible scaffold for biomedical applications such as tissue engineering, drug delivery and enzyme immobilization for bio sensing applications. Figure 15 shows the surface functionalization of PNFs by glutaraldehyde [81].

### 4.12. HNO_3_ (Nitric Acid)

Nitric acid (HNO_3_) is a colorless liquid with yellow or red fumes with an acrid odor. Cuervo et al. reported the functionalization of carbon nanofiber with HNO_3_. The oxidation of the nanofiber results in steric limitations of the adsorption. Both the capacity and the strength of adsorption decrease after the oxidant treatment of the carbon nanofibers, although in the case of chlorinated compound the specific component of the surface energy shows significant rise [82].

### 4.13. Stainless Steel

**Figure 16:**
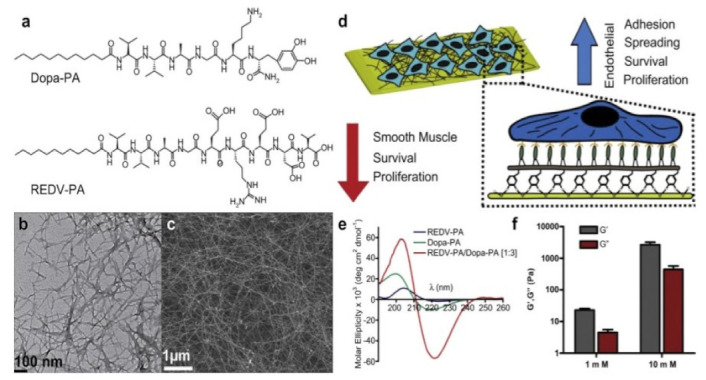
(**a**) Design and chemical representation of PA molecules. TEM (**b**) and SEM (**c**) images revealed the nanofibrous network that mimic the native matrix architecture. (**d**) Schematic representation of REDV-PA/Dopa-PA network, which is designed to functionalize stainless steel surface to support endothelial cell adhesion, spreading, viability and proliferation. (**e**) Circular dichroism results revealed formation of b-sheet structure, which drives nanofiber formation upon mixing Dopa-PA and REDV-PA at physiological pH. (**f**) Rheology results showed gelation as a result of nanofibrous network formation by Dopa-PA and REDV-PA at pH 7.4 [83]. Adapted with permission from Ref. [83]. 2011, Elsevier.

Stainless steels contain at least 10.5% chromium, less than 1.2% carbon and other alloying elements. Tekinay et al., reported the selective adhesion and growth of vascular epithelium cells on bioactive amide nanofiber functionalized with stainless-steel surface. Metal-based scaffolds comparable to tubings are the foremost most well-liked treatment ways for coronary disease. However, impaired endothelialization on the purple heart surface of the stents is also a serious limitation sometimes leading to harmful consequences inside the future. Coating the stent surface with relevant bioactive molecules is taken under consideration to help in recovery of epithelial tissue around the wound site. During this study, they developed self-assembled peptide nanofibers that mimic the native endothelium extracellular matrix and that are firmly immobilized on stainless steel surface through mussel-inspired adhesion mechanism. REDV Functionalization provided selective growth of epithelium cells on the stainless-steel surface. They synthesized dihydroxyphenylalanine-conjugated amide amphiphile and REDV-conjugated peptide amphiphile that are self-assembled at physiological pH. Researchers reported Dopa conjugation enabled nanofiber coating on chrome steel surface that is that the foremost typically used backbone of this stents (Figure 16) [83]. The strategy for surface biofunctionalization created a favourable microenvironment to market epithelium cell growth on stainless-steel surface, thereby providing an economical platform for bioactive tubing development for future treatment of cardiovascular diseases.

### 4.14. Zwitterionic Sulfobetaine

Sulfobetaine (SB) family is white powder solid at traditional temperature because of zwitterionic nature. They are wide utilized in cosmetics, supermolecule purification, protein separation, and protein solubilization. Wang et al. reported the zwitterionically surface functionalized PVA-co-PE nanofiber materials by click chemistry.

The functionalization of zwitterionic Sulfobetaine on surfaces of PVA-co-PE nanofiber membrane was performed. The functionalization of nanofibers with zwitterionic materials on surfaces stops the nonspecific adhesion of biomolecules and micro-organism are in demand for prime potency biosensors and affinity membranes.

**Figure 17:**
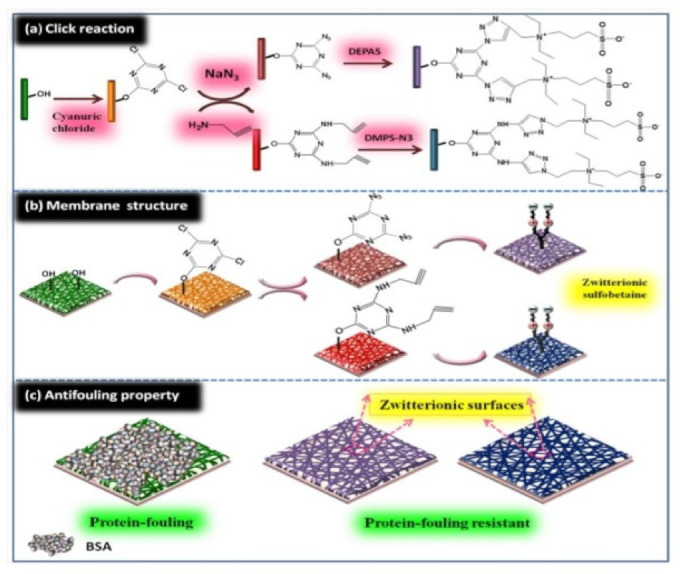
Zwitterionic sulfobetaine functionalization of PVA-co-PE nanofiber membranes and their nonspecific protein resistance performance [84]. Adapted with permission from Ref. [84]. 2013, Royal Society of Chemistry.

PVA-co-PE nanofiber membranes with zwitterionic showed significant protective performance by effectively resisting bovine supermolecule (BSA) protein sorption as shown in Figure 17 [84].

### 4.15. Weak Acid Cation-Exchange Ligand

Schneiderman et al., reported surface functionalization of electrospun carbon nanofiber mats with weak acid cation-exchange ligand. Surface functionalization results in increased binding capacity of lysozyme. These surface functionalized nanofibers have an application for adsorption or purification with high capacity and throughput [85].

### 4.16. Primary Amine

**Figure 18:**
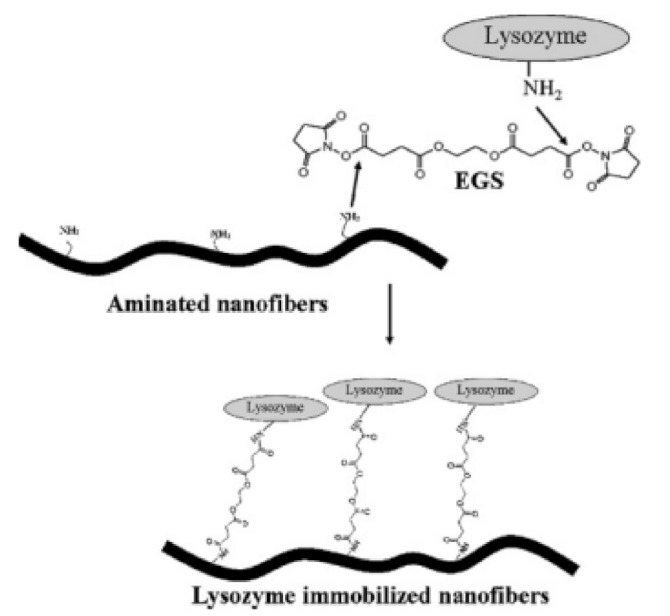
Schematic diagram of lysozyme immobilization process [86]. Adapted with permission from Ref. [86]. 2008, John Wiley and Sons.

Park et al., reported Surface Functionalization Electrospun nanofibers for immobilization of bioactive molecules, during this study the mixture of biodegradable poly (€-caprolactone) (PCL) and poly (D, L-dairy product-co-glycolic acid)-poly (ethylene glycol)-NH_2_ (PLGA-b-PEG-NH_2_) block polymer was electrospun to provide surface functionalized nanofibers. Resulting nanofibrous mesh was functionalized with primary amines and for immobilization of biomolecules lysozyme was used as model enzyme (Figure 18). Various biomolecule showed efficient immobilization after functionalization [86].

### 4.17. Beta-Cyclodextrins

**Figure 19:**
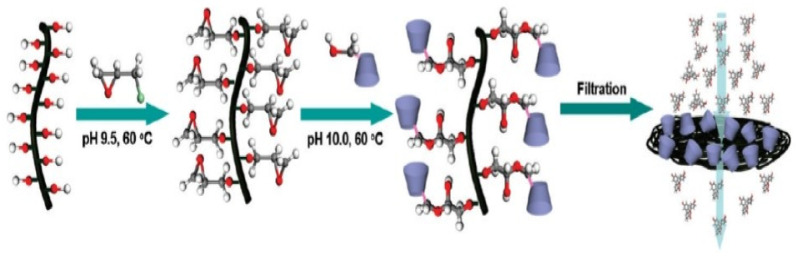
Schematic illustration of the functionalization of a carbonaceous nanofiber membrane by β-CD and molecular filtration application [87]. Adapted with permission from Ref. [87]. 2011, American Chemical Society.

β-Cyclodextrin (β-CD) have the cavity at the outer surface for several group groups, however hydrophobic within the cavity, thus β-CD is soluble in water. Such a characteristic has been wide applied inside the fields of controlled drug release, separation, and adsorption. Chen et al. reported carbonaceous nanofiber membrane functionalized by β-cyclodextrins. The fabricated and functionalized nanofibers showed potential application in molecular filtration. The membrane shows a noteworthy capability to function as an ideal molecular filter through complexation of acid-base indicator molecules with the cyclodextrin molecules grafted on the CNFs. Engineering the surface of this carboniferous nanofiber membrane might enable it to be used for different applications like chiral separation and drug delivery shows in Figure 19 [87].

### 4.18. COOH-Containing Polymer and TiCaPCON Film

Anton M. Manakhov et al., (2019), reported the functionalization of PCL nanofibers with COOH-containing compound using atmospheric pressure plasma copolymerization of CO_2_ and C_2_H_4_, and nanostructured TiCaPCON film by the technique of thermionic valve sputtering. The TiCaPCON-coated PCL nanofibers exhibited increased adhesion and proliferation of rate 3T3-E1 cells, promoted the formation of Ca-based mineralized layer SBF and resulted as promising material for bone tissue regeneration. The PCL-COOH nanofibers illustrate higher adhesion and proliferation of IAR-2 cells, that shows their high potential for skin reparation and wound dressing [88].

### 4.19. Glycine-Phenylalanine-Hydroxyproline-Glycine-Glutamate-Arginine (GEOGER) Peptide

Kolambkar et al., reported Nanofiber orientation and surface functionalization to modulate human mesenchymal somatic cell behaviour in vitro. Electrospun nanofiber meshes have emerged as a replacement of scaffold membranes for tissue regeneration. During this study, they investigated the results of nanofiber orientation and surface functionalization on human mesenchymal stem cell (hMSC) migration and osteogenic differentiation. Poly (€-caprolactone) meshes with bound topography, were generated by electrospinning aligned nanofibers on a rotating mandrel, whereas every way-oriented controls were accumulated on a stationary collector. Each aligned and random meshes were coated with a triple-helical, type-I collagen-mimetic peptide, containing the glycine-phenylalanine-hydroxyproline-glycine-glutamate-arginine (GEOGER) motif. Their results indicate that nanofiber GFOGER amide functionalization and orientation modulate cellular behavior, individually and in combination. Aligned nanofiber meshed displayed hyperbolic cell migration on the direction of fiber orientation compared to random meshes; however, fiber alignment did not influence osteogenic differentiation. GFOGER considerably enhanced the migration, proliferation and osteogenic differentiation of hMSCs on nanofiber meshes [89].

### 4.20. Polydopamine

Polydopamine (PDA) is a rising nature-inspired biopolymer material that possesses several captivating properties as well as self-assembly and universal adhesion. Xie et al., reported Mussel-inspired supermolecule-mediated surface functionalized electrospun nanofibers for pH-responsive drug delivery. This paper demonstrates that a mussel-inspired protein polydopamine coating will tune the loading and responsive rate of charged molecules from electrospun poly (€-caprolactone) (PCL) nanofibers in solutions with completely different pH scale values. In vitro release profiles show that the positive charged molecules release considerably quicker in acidic than those in neutral and basic environments. This pH-responsive drug delivery systems supported polydopamine-coated PCL nanofibers could have potential application at intervals the oral delivery of metastatic tumor medicine for treating stomachal cancer and in epithelial duct delivery of antiviral drugs [90].

### 4.21. Biotin

**Figure 20:**
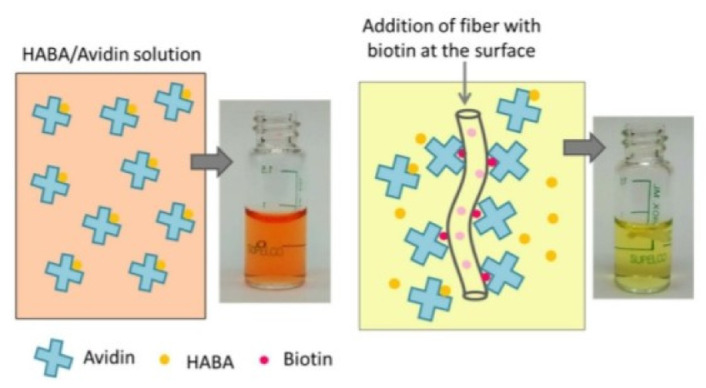
Illustrations and real pictures of the HABA/avidin solutions. Initially, HABA and avidin form a complex with a strong orange color (absorbs light at 500 nm). When a fiber containing biotin is added to the solution, avidin binds biotin due its higher affinity breaking the HABA/avidin complex and leading to a color change (decrease in the absorbance at 500 nm) [91]. Adapted with permission from Ref. [91].

Frey et al., reported surface modified Poly (lactic acid) electrospun nanofibers for biosensor applications. In this work, B-complex vitamin surface functionalized by hydrophilic non-water-soluble biocompatible Poly (lactic acid) (PLA) nanofibers are created for its potential use as biosensors. Variable concentration of biotin was incorporated into PLA fibers along with poly (lactic acid)-block poly (ethylene glycol) (PLA-b-PFG) block polymers. The incorporation of PLA-b-PEG block copolymers not only diminished fiber diameter however dramatically magnified the amount of biotin out there at the fiber surface able to bind avidin. Whereas biotin provided surface functionalization PLA-b-PFG provided hydrophilicity to the ultimate fibers. Finally, fiber water stability tests disclosed that each B-complex vitamin & PLA-b-PEG migrated to the liquid section when comparatively extended the periods of water exposure. The practical hydrophilic nanofiber generated during this work shows associate degree inherent utilization as a biosensor as shows in Figure 20 [91].

### 4.22. Poly (2-Methacryloyloxyethyl Phosphorylcholine) (Poly MPC)

**Figure 21:**
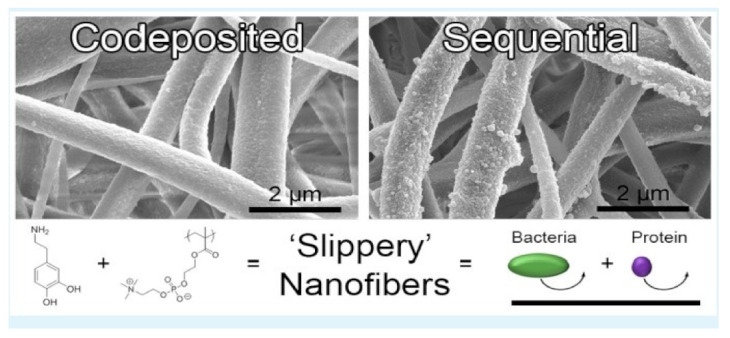
Antifouling electrospun nanofiber mats functionalized with polymer zwitterions [92]. Adapted with permission from Ref. [92]. 2016, American Chemical Society.

Poly (2-Methacryloyloxyethyl phosphorylcholine) (PMPC) is methacrylate-based compound with a zwitterionic phosphorylcholine moiety on the side chain. The phosphorylcholine moiety is like the head cluster of phospholipids in cell membranes. PMPC has been wide exploited throughout various applications, like protecting materials for water treatment, biotechnology, and nanomedicine. Schiffman et al. reported Antifouling Electrospun Nanofiber Mats Functionalized with zwitterionic compounds. This zwitterionic polymer coating accelerate the convenience of the zwitterion to expeditiously limit biofouling on nanofiber membranes. The study showed that, by decorating the surfaces of cellulose nanofiber mats with PolyMPC, we can make high performance nanofiber mats that hold potential applications in tissue engineering scaffolds and water purification technologies. Figure 21 shows protective electrospun nanofiber mats functionalized with zwitterions [92].

Table 3 is summarizing all the materials used for surface functionalization of nanofibers along with applications.

## 5. Applications of Surface Functionalized Nanofibers

The surface properties of any material/device can be modified via surface functionalization an efficient way to achieve specific goal such as producing a required bio response or hindering a potential undesirable reaction. Following are case studies which show the various applications of surface functionalization for various purposes such as wound healing, tissue engineering, anti-bacterial, anti-viral properties and various biomedical applications.

### 5.1. Wound Healing

**Figure 22:**
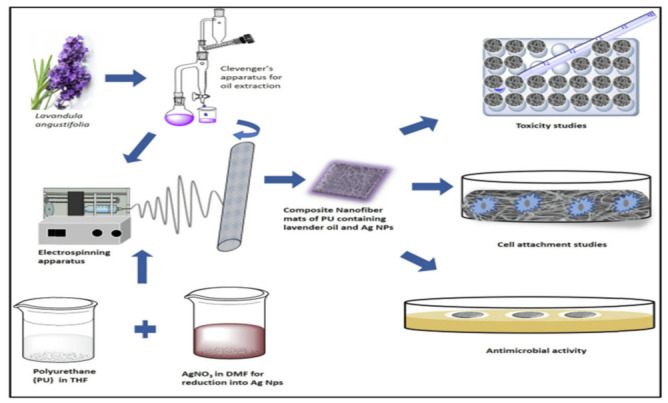
Schematic representation of the fabrication of PU/LO/Ag NPs nanofiber composites using the electrospinning technique to create multifunctional nanofibers that are excellent for cell growth and as an antimicrobial agent. PU = polyurethane, LO = lavender oil [93]. Adapted with permission from Ref. [93]. 2019, Elsevier.

Sofi et al., 2019 investigated exceptional antibacterial properties of lavender oil and silver loaded nanofibers against *E. coli* and *S. aureus*. The composite electrospun wound dressing nano fibers were prepared containing polyurethane encased with lavender oil and silver nano particles and characterized by Scanning Electron Microscopy (SEM), Transmission Electron Microscopy (TEM), Fourier transform infrared spectroscopy (FTIR) and X-ray diffractometer (XRD) for chemical analysis of nanofibers. It was observed that after lavender oil and silver loading onto polyurethane nanofibers, there is increased in hydrophilicity, especially with lavender oil which decreases the rigidity of the polyurethane fibers through diffusion and permeation. For this they first prepared silver nano particles by reducing AgNO_3_ salt by aging it in N, N-dimethylformamide (DMF) (20 parts by weight of solvent) and Lavender oil was obtained from *Lavandula angustifolia* by the extraction. After nano fiber preparation with silver nanoparticles and Lavender oil, it was further analyzed for various cytotoxicity tests, wetting properties and antibacterial activities in that the Lavender oil and silver nanoparticles loaded nano fibers were shown as an effective anti-bacterial wound dressing material because of its increased hydrophilicity, bactericidal activity (against *S. aureus* and *E. coli*) and increased biocompatibility of composites (Figure 22) [93].

Sana Ullah et al., used silver sulfadiazine (AgSD) for surface functionalization of polyacrylonitrile nanofiber (PAN) mats. They used silver sulfadiazine (AgSD) for surface functionalization because of its most efficient antibacterial activity, especially in burn wound dressing. Silver has a potential antibacterial activity, which can be used for better effect in wound healing. It was detected that an increase in antibacterial activity of nanofibers with increased in concentration of AgSD [94].

**Figure 23:**
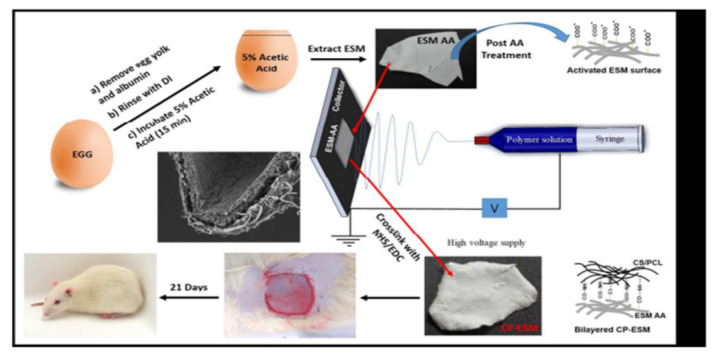
Surface modification of eggshell membrane with electrospun chitosan/polycaprolactone nanofibers for enhanced dermal wound healing [95]. Adapted with permission from Ref. [95]. 2018, American Chemical Society.

Preetam and colleagues modified eggshell membrane using chitosan/polycaprolactone (CS/PCL) nanofibers for better application in wound healing. Egg shell membrane (ESM) primarily composed of protein (80–85%) and 10% is collagen (Type I, V and X) and remaining 70–75% is other proteins and glycoproteins. ESM is potential biomaterial for tissue engineering application because of its ability to imitate the extracellular matrix (ECM) with its unique chemical composition and also, because of its availability, biocompatibility and contaminant free material. This is for first time, reported as a natural shell membrane can be used as a biomaterial as a dressing in the burn wound healing. After in vitro and in vivo studies of these surface functionalized nanofiber it, was observed that within 14 days there was a 90% wound closure. And, after 4 weeks, complete and excellent wound healing was observed with dermal regeneration and re-epithelization. Hence it was acknowledged that modified ESM using chitosan nanofibers can be used in wound or burn healing because of its excellent results (Figure 23) [95].

Miao et al., fabricated anti-infective bandages using lysostaphin. Lysostaphin is an enzyme having definite bactericidal activity against *S. aureus*, which is immobilized on to biocompatible fiber of cellulose, i.e., cellulose-chitosan. The lysostaphin functionalized cellulose fibers showed an activity against the *S. aureus* in an in vitro skin model which is obtained after processed as a bandage preparation. Moreover, it showed low toxicity towards keratinocytes, which will suggest its good compatibility and hence it can be a good antimicrobial material in wound healing. In this study, they prepared cellulose fibers by electrospinning solution of cellulose, cellulose-chitosan and cellulose poly (methylmethacrylate). These all are tested for the tensile strength testing, which showed reasonable mechanical strength (6 +/− 0.5 MPa) for oxidized cellulose fiber mats and tested for antimicrobial activity against *S. aureus* from which it is observed that lysostaphin-based cellulose fibers showed complete counteract of *S. aureus* population and hence it can be considered as an anti-infective, antistaphylococcal, biocompatible and stable bandages for wound healing application [96].

Sudipto Pal et al., fabricated bacterial cellulose (BC) functionalized with silver nanoparticles. They synthesized silver nanoparticles inside the porous 3-dimentional weblike bacterial cellulose network by using UV light irradiation. Photochemically, AgNPs where deposited onto the BC gel network and they were bonded chemically to the surfaces of cellulose fiber. These hybrid composites were tested for antibacterial activity against the gram-negative bacteria (*E. coli*) by disk diffusion and growth dynamics methods which shown high bacteria killing performance. The Ag/BC pellicles did not exhibited substantial quantity of silver release after a long soaking time [97].

### 5.2. Drug Delivery

Electrospinning is a versatile technique for fabrication of synthetic, natural, and hybrid materials into polymer nanofibers. The electrospun nanofibers possess high surface to volume ratio which can improve drug loading, cell attachment, and mass transfer properties to develop into drug delivery system. Following are some case studies of drug delivery, showing the application of surface functionalized nanofibers in formulating an effective and potential drug delivery system used to modify the release kinetics, biodistribution and to minimize toxic effects, with some enhancement in the therapeutic index of a drug. Controlled drug delivery system (CDDS) has a wide application in treating various diseases however nano materials emerged as a new generation of CDDS, which can be used to modify the release kinetics, biodistribution and to minimize toxic effects, with some enhancement in the therapeutic index of a drug. Jiang et al., demonstrated the enhanced loading and release rate of charged molecules from electro spun poly(-caprolactone) nanofibers in solutions of different pH values due to mussel-inspired protein polydopamine coating. The fibers were prepared by standard electrospinning by dissolving PCL (polycaprolactone) in a solvent mixture consisting of dichloromethane and N, N-dimethylformamide and polydopamine was coated on fiber membrane after preparation. The prepared nanofibers also tested for cytotoxicity test which indicated remarkably decreased cell viability due to treatment with DOX-(Doxorubicin) released solutions which substantiated pH responsive release of DOX from PDPCL nanofibers and toxicology data. This study concluded that air-plasm treated PCL and PLA (poly lactic acid) nanofibers can be used as a carrier for loading and releasing the charged molecules in pH-responsive way. Besides, modified pH-responsive loading kinetics and release of charged molecules was observed due to mussel-inspired protein polydopamine coating. The novel nano formulation may exhibit possible application in drug delivery to precise targets related with pH variations [90].

Electric field to produce electric charge jets from polymer solution or melts which on drying by means of evaporation of solvent produce nanaofibers. In sustained release drug delivery nanofiber are prepared by incorporated the drug into the nanofiber matrix, a drug must be properly encapsulated into nanofiber structure. Many researchers work on these crucial areas which show effective drug release profile of the various drugs like, itraconazole, tetracycline hydrochloride and doxorubicin hydrochloride, etc.

Chen et al., developed nanofiber by using potential carrier for sustained release of polycaprolactone (PCL)/polylactic acid (PLA) core-shell porous drug-carrying nanofibers by using coaxial electrospinning technology. Roxithromycin (ROX), an antibacterial agent was encapsulated in core layer. It exhibits good antimicrobial activity against *Staphylococcus aureus*, it showed that the release rate of the prepared coaxial porous nanofibers with two different pore sizes was 30.10 ± 3.51% and 35.04 ± 1.98% in the first 30 min, and became 92.66 ± 3.13% and 88.94 ± 1.58% after 14 days [98].

Topur and Uyar summarized the use of cyclodextrin as an ideal drug carrier for drug delivery application because it is natural, edible, and biocompatible material. It is having both the hydrophilic and hydrophobic characteristics for complexation of drug and its solubility, respectively. Drug molecules are protected from physiological degradation and/or elimination resulting in increased stability and bioavailability of drug by complexation with cyclodextrin. An advanced drug delivery requires non-toxic, biocompatible and stable pharmaceutical material to deliver greater drug loading and keeps the drug stable and active, for this Topur and Uyar suggested that the cyclodextrin-functional nanofibers will be suitable material for drug delivery application [99].

### 5.3. Bacterial and Viral Pathogen Detection

Using surface functionalization, it is also possible to detect bacterial and viral pathogen. Following is mentioned case study show the application of surface functionalization in bacterial and viral pathogen detection.

Yilun Luo et al., reported the electrospun nitrocellulose nanofibrous membrane and its antibody functionalization for bacterial and viral pathogen detection. The capillary action of the nanofibrous membrane was enhanced through oxygen plasma treatment. An electrospun biosensor was designed using capillary separation and conductometric immunoassay. Spray deposition method was used to fabricate the silver electrode, which is non-invasive for electrospun nanofibers. Confocal laser scanning microscope (CLSM) and scanning electron microscope was used to verify antibody attachment and pathogen binding effect. Glutaraldehyde was used as a cross linking agent to attach antibody to electrospun fiber mats and for protein binding enhancement and reduce pH effect. The electrospun biosensors tested on *E. coli* O157: H7 and bovine viral diarrhea virus (BDVD) samples. In which the electrospun biosensors was responded for both the samples (*E. coli* O157: H7 and BDVD) used. The detection time was 8 min and detection limit were 61 CFU/mL and 10^3^ CCID/mL for bacterial and viral samples respectively [100].

### 5.4. Tissue Engineering and Regenerative Medicine

Currently, Electrospun nanofibers received great attention because of their potential applications in biomedical devices, tissue engineering scaffolds, and drug delivery carriers which can be attributed to their superior properties.

Ma et al., developed surface modified electrospun poly-(caprolactone) (PCL) nanofibers in order to make it compatible with endothelial cells and extend its use as a blood vessel tissue-engineering scaffold. Air plasma treatment of PCL nanofiber was given to implant gelatine, in which -COOH group was introduced and followed by covalent grafting of gelatine molecule, by using water soluble Carbodimide as a coupling agent. X-ray photoelectrospectry and colorimetric methods were used to identify any chemical change in material surface at the time of surface modification. Endothelial cells were cultivated to evaluate the cytocompatibility of surface modified PCL nanofiber and aligned PCL nanofiber. After study it was observed that gelatin grafting significantly improved endothelial cell spreading and growth on PCL nanofiber [69].

The renewal of lost, injured, or aging cells and tissues nearly to their innate function is the prime role of regenerative medicine which lead to important expansions in biomedical research and clinical practice. Its applications have been extended to diverse fields comprising cell therapy, diagnosis, tissue engineering, drug delivery and gene delivery owing to ability to alter at molecular level and the use of several functionalized nanomaterials.

Massoumi et al., express functionalization of graphene oxide (GO) with poly (2-hydroxymethyl methacrylate)-graft-poly (caprolactone) [P (HEM-g-CL)] using polymerization approach and fabricated electrospun nanofibers with gelatine. The authors synthesized GO by oxidizing pristine graphite powder and the incorporation of acetyl chloride into it, to offer an atom transfer radical polymerization (ATRP) microinitiator (GO-CL). With the help of gelatine, uniform, conductive and biocompatible nanofibers were prepared by electrospinning which were investigated for its various properties. The biocompatibility of this electrospun nanofibers were confirmed by assessing survival rate of human osteoblast MG-63 cells on nanofibers using MTT assay. Also, the electrospun nanofibers were analyzed for electrical conductivity which were found to be proper conductive for scaffolding. Authors reported simple, convenient and effective approach to preparation of GO-g-[P(HEM-g-CL)]/gelatine electrospun nanofibers with adequate biocompatibility, biodegradability and conductivity for different applications [101].

### 5.5. Targeting Strategies

An application of surface functionalization in planning some targeted strategies such as tumor targeting has been explored. Patil and his associates reported versatile surface functionalization technique for polymeric nanoparticles. The fact is when a dipole co-polymer, polymeric ethylene glycol (PLA-PEG) is introduced in the oil/water emulsion which is used in the polymeric nanoparticle formulation, PLA blocks the partitions into polymer containing organic phase while PEG blocks partitions into aqueous phase. Nanoparticles with PEG on the surface can be obtained after desolvation. The study facilitates IAASE approach, by simultaneous incorporation of biotin and folic acid which is known as tumor targeting ligands on drug loaded nanoparticles, which was confirmed by NMR, TEM, surface plasmon resonance and tumor cell uptake studies. Authors claimed that the new surface functionalization technique enables the development of targeted strategies employing multiple ligands on a single surface to target a particular tissue [102].

### 5.6. Anti-Bacterial Application

Surface functionalization a simple technique can be used to functionalize the surface of nanofibers to enhance number of activities like antibacterial, antimicrobial, for developing targeted strategies, etc. Following is the case study related with applications of surface functionalization in increased anti-bacterial activity.

Nirmala et al., prepared and characterized chitosan blended polyamide-6 nano fibers for human osteoblastic (HOB) cell culture applications, which was prepared by electrospinning process. In this study, they used chitosan which is a natural biopolymer which is non-toxic, possessing polycationic chelating and film forming properties, because of its active amino and hydroxy functional group.

Chitosan is used because of its biodegradability, biocompatibility, non-toxicity, antimicrobial activity, and absorption potentiality. Also, an in vitro cytotoxicity study reveals that polyamide-6/chitosan blended nanofibers were non-toxic for the osteoblastic cell culture and their characterization using FT-IR spectroscopy indicates its increased stability due to amide group terminated polyamide-6 nanofibers and also it is observed that the bands were corresponding to amide II and amide III be decreased with increasing chitosan content [103,104].

### 5.7. Biomedical Application

The nanofiber technology become prevalent to overcome recent challenges in the biomedical field viz. burn and wound care, organ repair and treatment of various diseases. Even nanofibers can be engineered into advanced macro-scale structures. Moreover, activity and physicochemical properties of nanofibers could be enhanced via surface functionalization for biomedical applications, diagnosis, and targeted drug delivery.

Wang and associates reported aminopropyltriethoxysilane (APTS)-mediated surface modification of nanohydroxyapatite employing various surface functional groups for potential biomedical applications. Authors tried to modulate nanohydroxyapatite surface with APTS and APTS amine groups which was further altered with acetic anhydride and succinic anhydride to produce neutral surface charge and nanohydroxyapatite particles with negative charged. These nanohydroxyapatite derivatives were further characterized and evaluated for cytotoxicity with the help of MTT [3-(4,5-dimethyl thiazol-2-yl)-2,5 diphenyl tetrazolium bromide] colorimetric assay of mouse fibroblasts (L929 cells). For these functionalized derivatives, in vitro hemolysis assay assessed for the determination of blood compatibility. The present research can be used for development of nanohydroxyapatite based composite materials for different biomedical application [105].

#### 5.7.1. Immobilization of Bioactive Molecules

Nanofibers have various application like immobilization of bioactive molecule. Electrospun nanofibers have been widely utilized as support materials for enzyme immobilization due to their various merits, including large specific surface area, high porosity, and interconnectivity which in turn helps in crosslinking, covalent coupling, encapsulation, etc.

Kim et al., synthesized nanofiber by blend of the biodegradable poly (e-caprolactone) (PCL)and poly (D,L-lactic-co-glycolic acid)-poly(ethylene glycol)-NH_2_ (PLGA-b-PEG-NH_2_) block copolymer was used to produce surface functionalized nanofiber. The synthesized nanofiber was prepared by primary amine on surface using lysoenzyme as a model enzyme. It immobilized lysozyme on its surface with improved activity due to its huge surface area [86].

#### 5.7.2. Chemotherapy-Cancer Theranostics Application

Electrospun nanofibers revealed as capable approach for implantable stage in cancer treatment, on-site delivery of therapeutic compounds and marginal adverse effect to normal tissue. 

Xu et al., developed BCNU—loaded implantable functionalized electrospun nanofiber for cancer treatment by using poly (ethylene-glycol)-poly (Lactic acid) (PEG-PLLA) diblock copolymer with controlled release of 1,3-bis(2-chloroethyl)-1 nitrourea(BCNU). ESEM (Environmental scanning electron Microscope) showed very smooth and uniformed BCNU-Loaded PEG-LLA fiber formation. The resulting nanofibers have antitumor activity with controlled release effect, and it is suitable for postoperative chemotherapy of cancer.

#### 5.7.3. Implantable Smart Magnetic Nanofiber

Magnetic nanofibers are commonly used in various application like cancer treatment which responds to external magnetic field. As magnetic NPs can be precisely implanted in the tumor site which provides high loading and superior magnetic response. 

Susikala et al., reported implantable smart magnetic nanofiber for endoscopic hyperthermia treatment and tumor-triggered controlled drug release prepared using two component smart nanofiber matrix from mono disperse iron oxide nanoparticle (IONPs) and bortezmib (BTZ) as chemotherapeutic agent. The mussel-inspired surface functionalization using 2-(3,4-dihydroxyphenyl) ethylamine (dopamine) was done to conjugate the borate-containing BTZ anticancer drug through a catechol metal binding in pH-sensitive manner. It is applicable in both endoscopic hyperthermia and triggered controlled dung release. The functionalizes have an advantage due to low toxicity toward normal cell and highly effective in consideration of therapeutic efficacy [106].

#### 5.7.4. Liver Cancer Therapy

Cancer is already considered as the lethal disease in the world. It has been reported that liver cancer caused 466,100 new cases and 422,100 deaths in China alone in 2015. Chemotherapy is the most usual approach for treatment of liver cancer [107].

Wu et al., fabricated the PTX -loaded mesoporous hollow SnO_2_ nanofiber conjugated with folic acid. According to characterization results of SEM and TEM showed hollow structure of average outer diameter of 200 nm and wall thickness was 500 nm. The rate of drug release and dissolution profile of PTX was significantly improved. In vivo and in vitro experimentation showed the efficient inhibiting effect on the growth of liver cancer cells [107].

### 5.8. Cell Culture Application

Rieger et al., work on the concept of mat hydrophilicity and surface chemistry by polyelectrolyte functionalization nanofiber to control the collection and inactivation of *E. coli*. The collection and inactivation of Escherichia coli K12 was examined using cellulose nanofiber mats that were surface-functionalized using three polyelectrolytes: poly (acrylic acid) (PAA), chitosan (CS), and polydiallyldimethylammonium chloride (pDADMAC). Hydrophilicity paired with neutral or positive charge improved the collection of *E. coli* K12, whereas hydrophilic cationic nanofiber mats exhibited the highest killing of *E. coli* K12 [79].

Nanofiber plays the promising approach in the cell culture design. The morphological features of nanofibers simulate extracellular matrix that is in vivo microenvironment hence these are favorable substrates for regenerative medicine. Nanofibers used for the culture including human pluripotent stem cells known as oligopeptide-immobilized nanofibers. These are fabricated by employing synthetic or natural polymers, and ECM-modified nanofiber.

Nirmala et al., developed chitosan blended polyamide-6 nanofibers by a via electrospinning process for human osteoblastic (HOB) cell culture applications using novel single solvent system. FT-IR and SEM showed the effective result, i.e., proper adhesion, viability and proliferation properties of osteoblast on the polyamide-6/chitosan blended nanofibers were analyzed by in vitro cell compatibility test [102].

### 5.9. Anticoagulant Activity

Li juanu et al., developed nanofiber functionalized magnetically responsive heparin—immobilized cellulose nanofiber composites were synthesized by wet—wet electrospining method by using—1-methyl-3-methyl imidazolin acelate ((EMIM)AC) into an aqueous coagulation bath Synthetic fiber developed in three types of nano composite fiber. These nano composite fibers were characterized by Thermogravimetric analysis, Liquid chromatography mass spectrometer fourier transform infra-red X-ray diffraction Spectroscopy. It showed anticogulant activity [108].

Table 4 is illustrating various biomedical applications of surface functionalized nanofibers.

## 6. Toxicity Study of Surface Functionalized Nanofiber

Toxicity study is most important and, critical aspect for assessment of lethal dose and, noxious effect of surface functionalized nanofiber. In study report this can be determined by In vitro, In vivo assay. In vitro hemocompatibility of nanofiber can be examined by the hemolysis and cell viability assay to confirm the safety as well as biocompatibility of functionalized nanofiber material. MTT assay used for the assessment of metabolic activity of cells [109,110]. Activated partial thromboplastin time (APTT) assay and Prothrombin time (PT) assay analyze for the clotting measurement in electrospun nanofibers. Protein absorption or concentration on surface functionalization scaffold can be analyzed by the Bicinchoninic Acid (BCA), Fibrinogen adsorption assay, Bradford assay, lowry assay, and biuret reaction, etc. reported in study report [111,112,113]. Deposition of platelet on functionalized nanofiber performed by the help of platelet adhesion or activation assay. However, deposition of minerals on the surface determined by mineralization assay, by using ARS staining. Antibacterial activity of drug loaded nanofiber scaffold used the in the wound healing application determine by the In vitro antibacterial assay [114]. According to study report cells used in the assay to prove the biocompatibility, cyto-toxicity, as well as to examine surface functionalization approach of nanofiber. In the literature report author fabricate a MEH-PPV (2-methoxy-5-(2-ethylhexyloxy)-1,4-phenylenevinylene) and PCL (Polycaprolactone) based electrically conductive scaffold for neural tissue regeneration activity and, scaffold surface functionalized by the help of 3-aminopropyltriethoxysilane (APTES) and 1,6-hexanediamine (HDA) for cell adhesion. In this study author performs several In vitro assay for assessment of toxicity. MTS cell assay shows the impression surface functionalization on cytocompatibility. Various types of cells behavior activity also perform such as live−dead assay for the impact of functionalization on scaffold cell adhesion, Beta(III) Tubulin Immunochemistry to confirm the MEH:PCL scaffold for neural tissue differentiation. Electrically conductive test also confirms the neurite differentiation. Finally, author concluded that Surface amination is the best approach to for MEH-PPV:PCL nanofiber as compared to conventional expensive coating of biomolecule for neural tissue regeneration activity [115].

## 7. Conclusions

Nanofibers are having broad spectrum biomedical, healthcare and theranostic applications. The nanotechnology is becoming prime choice of drug delivery across the globe due to its efficiency in drug delivery and disease targeting. The nanofibers are one of the prime carriers used across the world and contributing to the large extent to global pharmaceutical market. The surface functionalized nanofiber is the preferred choice of drug delivery in various infections, diseases, and disorders. The recent advancements in surface functionalization techniques are exploring the scope of multiple biomedical applications. In the future, the hybridization of two or more surface functionalization techniques for nanofibers may result in efficient drug delivery. The large scope of nanofiber technology in healthcare may result in improved global importance of nanofibers with large significant contribution to the economy of the pharma sector.

## Figures and Tables

**Table 1:** Fabrication techniques of nanofibers (Adapted from Ref. [6]).

Fabrication Technique	Advantages	Disadvantages
Drawing	Simple equipment	Discontinuous process Not scalable No control on fiber dimensions
Template synthesis	Continuous process Fiber dimensions can be varied using different templates.	Not scalable
Temperature-induced phase separation	Simple equipment Convenient to process Mechanical properties of the fiber matrices can be varied by changing the polymer composition	Limited to specific polymers Not scalable No control on fiber dimensions
Molecular self- assembly	Only smaller nanofibers of few nm in diameter and few microns in length can be fabricated	Complex process Not scalable No control on fiber dimensions
Electrospinning	Simple instrument Continuous process Cost effective compared to other existing methods Scalable Ability to fabricate fiber diameters Few nm to several microns.	Jet instability Toxic solvents Packaging, shipping, handling

**Table 2:** Nanofiber modification techniques Adapted with permission from Ref. [47].

Methods of Modification	Advantages	Disadvantages
Physical blends	Direct and easy Uniform functionalization	Burst release Only limited amount of functional group on the surface of nanofiber Functionality must survive processing
Core-shell electrospinning	Functional components could be encapsulated in the core of fibers Sustained release	Complex process Strict conditions to produce core-shell structured nanofibers Functionality must survive processing
Post functionalization Plasma treatments Wet chemical etching Click chemistry Surface graft	Surface functionalization Many choices could be selected Tolerant to more sensitive functional molecules	Need to process after electrospinning Need relative strict condition without compromising nanofiber structure

**Table 3:** Materials used for surface functionalization of nanofibers.

Sr. No.	Surface Functionalized Nanofibers	Material Used for Surface Functionalization	Applications	References
1	Polyamide 6/O-MMT composite nanofibers by Fe_2_O_3_ sputter coating	Fe_2_O_3_ (Ferrous oxide/Iron oxide)	Improved thermal stability properties of composite nanofibers	[68]
2	Gelatin grafted Electrospun Poly (caprolactone) nanofibers	Gelatin	Blood vessel tissue engineering	[69]
3	Size controlled silver Nanoparticle coated nanofibers	Silver	Wound Dressing	[70,71]
4	Surface modified polycaprolactone electrospun nanofiber	Plasma treatment (ar or O_2_ gas)	Cell morphology, cell adhesion and proliferation study	[72]
5	Graphene oxide-silver nanocomposites functionalized biopolymer nanofiber mats	Graphene Oxide-silver	Antimicrobial	[73]
6	PAN nanofibers with zno-Ag heterostructure nanoparticle	Zno-Ag	Anti-Bacterial	[74,75]
7	Bioinspired surface functionalized electrospun polycaprolactone nanofibers	Nano-hydroxyapatite (nha)	Bone tissue engineering	[57,76]
8	PCL-collagen nanofibers	Collagen Coating	Tissue engineering	[78]
9	Polyelectrolyte functionalized nanofiber mats	Polyelectrolyte: Poly (acrylic acid) (PAA), Chitosan (CS) and polydiallyl dimethyl ammonium chloride (pdadmac)	Anti-microbial (Collection and inactivation of *E. coli*)	[79]
10	Surface functionalization of dual growth factor on hydroxyapatite-coated nanofibers for bone tissue engineering	Avidin-Biotin	Bone tissue engineering	[80]
11	Surface functionalized Polyaniline nanofiber	Glutaraldehyde	Bio-sensor	[81]
12	Carbon nanofiber functionalized with volatile organic compounds	HNO_3_ (Nitric Acid)	Improved adsorption properties	[82]
13	Peptide nanofiber functionalized ith stainless steel	Stainless Steel	Treatment of cardiovascular diseases	[83]
14	Surface (zwittenrionically) functionalized PVA-co-PE nanofiber materials by click chemistry	Zwitterionic Sulfobetaine	Antifouling performance	[84]
15	Surface—functionalized Electrospun carbon nanofiber mats	Weak acid cation-exchange ligand	Protein adsorption/purification and bio-separation	[85]
16	Surface functionalized electrospun biodegradable nanofibers	Primary amine	Immobilization of bioactive molecules	[86]
17	Carbonaceous nanofiber membrane functionalized by beta-cyclodextrins for molecular filtration	Beta-cyclodextrins	Molecular Filtration, Chiral Separation and drug delivery	[87]
18	Carbonaceous Nanofiber Membrane Functionalized by beta-cyclodextrins for Molecular Filtration	COOH-containing polymer and ticapcon film	Skin reparation and wound dressing	[88]
19	Surface functionalized nanofibers	Glycine-phenylalanine-hydroxyproline-glycine-glutamate-arginine (GEOGER) peptide	Tissue Regeneration	[89]
20	Mussel-inspired protein-mediated surface functionalized electrospun nanofibers	Polydopamine	pH-responsive drug delivery	[90]
21	Surface functionalized Poly (lactic acid) electrospun nanofibers for biosensor applications	Biotin	Bio-sensor	[91]
22	Antifouling electrospun nanofiber mats Functionalized with polymer zwitterions	Poly (2-methacryloyloxyethyl phosphorylcholine) (poly MPC)	Tissue engineering and water purification	[92]

**Table 4:** Biomedical applications of surface functionalized nanofibers.

Description of Surface Functionalized Nanofibers	Application	Reference
Electrospun PCL per nanofiber with bioactive nano-hydroxy apatite (nHA) using dopamine effective bioadhesive agent	Improving Osteogenesis	[57]
Gelatin grafted poly-(caprolactone) (PCL) nanofibers	Tissue Engineering	[69]
Cellulose nanofiber mats surface functionalized using three polyelectrolytes: poly (acrylic acid) (PAA), chitosan (CS), and polydiallyldimethylammonium chloride (pDADMAC)	Control the Collection and Inactivation of Escherichia coli	[79]
Biodegradable poly (e-caprolactone) (PCL) and poly (D,L-lactic-co-glycolic acid)-poly(ethylene glycol)-NH2 (PLGA-b-PEG-NH2) block copolymernanofibers	Immobilization of Bioactive Molecules	[86]
Lavender oil and Silver loaded nanofibers	Wound Healing	[93]
Silver sulfadiazine (AgSD) loaded polyacrylonitrile nanofiber	Antibacterial activity	[94]
Modified eggshell membrane using chitosan/poly-caprolactone (CS/PCL) nanofibers	Wound healing	[95]
Lysostaphin functionalized cellulose fibers	Antimicrobial material in wound healing	[96]
Bacterial cellulose (BC) functionalized with silver nanofibers.	wound healing (Antibacterial activity against the gram-negative bacteria)	[97]
N, N-dimethylformamide and polydopamine loaded poly (caprolactone) nanofibers	Drug Delivery	[90]
Polycaprolactone (PCL)/polylactic acid (PLA) core-shell porous drug-carrying nanofibers	Sustained release drug application	[98]
Cyclodextrin-functional nanofibers	Drug Delivery	[99]
Glutaraldehyde cross linked nitrocellulose nanofiber	Bacterial and viral pathogen detection	[100]
Graphiene oxide (GO) with poly (2-hydroxymethyl methacrylate)-graft-poly (caprolactone) [P (HEM-g-CL)] using polymerization approach and fabricated electrospun nanofibers with gelatine	Application in Regenerative Medicine	[101]
Polymeric ethylene glycol (PLA-PEG) loaded nanofibers	Targeted Strategies	[102]
Chitosan blended poly-amide-6 nano fibers	Human osteoblastic (HOB) cell culture application	[103,104]
Aminopropyltriethoxysilane (APTS)-mediated surface modification of nanohydroxyapatite	Different biomedical applications	[105]
The mussel-inspired surface 1191 functionalization using 2-(3,4-dihydroxyphenyl) ethylamine (dopamine) to conjugate the 1192 borate-containing BTZ anticancer drug	Implantable Smart Magnetic Nanofiber	[106]
PTX-loaded mesoperous hollow SnO2 nanofiber conjugated with folic acid	Liver cancer therapy	[107]
Magnetically responsive heparin—immobilized cellulose nanofiber	Tissue Engineering	[108]

## Data Availability

Not applicable.
